# Book Reviews

**Published:** 1823-05

**Authors:** 


### CRITICAL ANALYSIS
OF
### ENGLISH AND FOREIGN LITERATURE
RELATIVE TO THE VARIOUS BRANCHES OF
### Medical Science.

Quæ laudanda forent, et quæ culpanda, vicissim

Illa, prius, cretâ; mox hæc, carbone, *notamus*.—PERSIUS.

### DIVISION I.

### ENGLISH.

ART. I.—*Practical Observations on the Treatment and Cure of several Varieties of Pulmonary Consumption; and on the Effects of the Vapour of Boiling Tar in that Disease.* By Sir ALEXANDER CRICHTON, M.D. F.R.S. Physician in ordinary to their Imperial Majesties the Emperor and Dowager Empress of Russia, and to his Royal Highness the Duke of Cambridge; Knight Grand Cross of the second Order of St. Vladimir, Knight of the Red Eagle of Prussia of the second Class, &c.—8vo. pp. xxxvi, 261. Lloyd and Son, London. 1823.

[Concluded from page 336.]

THE third, fourth, fifth, sixth, and seventh chapters, are occupied with discussions regarding the various forms of consumption: of these, the "tubercular" stands first on the list. The author begins his account of this by a minute description of the tubercles, taken from the works of BAILLIE, BAYLE, and LAENNEC, which we must crave permission to omit, and pass on to the valuable practical remarks which follow. Sir Alexander Crichton, along with many other distinguished physicians, condemns the idea of making weakly children "hardy," by the system so frequently adopted of clothing them lightly, exposing them to the vicissitudes of the weather, and cold-bathing. With regard to this last, he observes, that "a strumous or scrofulous constitution is always increased by languid circulation; and, in those who are very weak, the chill produced by cold-bathing, instead of being immediately followed by heat and animation, not only continues for a considerable time after the patient quits the bath, but is succeeded by cold extremities, palpitation of the heart, contracted features, head-ache, dyspnœa, and every symptom of internal congestion and a want of a free circulation." When a child of strumous constitution becomes languid, with loss of appetite, and a "pinched" appearance of the features, it is in general a proof of scrofulous action having commenced. It is, however, during the changes in the system at the period of puberty, that tubercles of the lungs are most apt to be developed; and this is greatly facilitated by every form of privation or excess. The dyspnœa, dry cough, and emaciation, with fever, are sufficiently characteristic; and, as the author observes, no time is to be lost, if the interference of art is to be employed with any rational prospect of permanent benefit.

"Whoever has had much experience with the consumptive, and has been an attentive observer, must have remarked two very distinct classes of tubercular consumption: one of a slow and insidious progress, accompanied by all the decided marks of phthisis, except a quick pulse and daily symptomatic fever; the other characterized by hectic fever, with daily exacerbations. These are not stages of one and the same disease; for although the first, which may be called the chronic kind, is accompanied at last with hectic fever, yet the symptoms of tubercular phthisis may continue making slow progress for one or two, or even several years, before this event occurs; whereas the acute kind of tubercular phthisis is accompanied with a quick pulse and daily exacerbations of fever from almost the beginning of the complaint, and, in comparison with the other kind, runs a very rapid course. The difference between these two kinds of tubercular consumption is not in degree, but in the constitution of the patients and the state of the lungs.

"In the chronic kind, the formation of fresh tubercles goes on slowly; and the enlargement of each tubercular mass, and its progress in softening, is also slow, and unaccompanied by inflammation. When one bag of softened tubercular matter has been evacuated by coughing, the patient apparently recovers a little, the frequency of the cough is sensibly diminished, and there is a general improvement in his health for a short time: he has a kind of fallacious respite, which is often attributed to the operation of medicine, which, if judiciously applied, certainly does assist nature in her operations. In a certain lapse of days, weeks, or even months, the dyspnœa increases; the patient has bad nights; his strength fails; and at last there is a fresh attack of cough, with the mixed expectoration peculiar to tubercular consumption. Of those who recover, I have known some who have struggled in this manner for nearly a year before they could be said to be in a state of uniform convalescence, and consequently before the greater number of tubercular masses were resolved. In women, the non-appearance of the catamenia, which is always a concomitant of an advanced phthisis, does not occur in this chronic kind until the last periods of emaciation; whereas, in the acute kind, it generally occurs soon, the *rationale* of which is too evident to require explanation.

"The prognosis in this variety of phthisis is certainly unfavourable, like that of all the varieties of pulmonary consumption; but my opinion is, that rather more than a tenth part of those who are attacked with it may be saved if the disease be taken in time, and the most watchful attention be paid to every circumstance of the case and its various changes."

Patients labouring under this form of phthisis are, of course, to be confined rigidly to the house during the winter; but the author, not content with this, enjoins their being almost equally so in summer, unless the atmosphere be very warm and calm. With regard to diet,—"all tea and coffee, and acid drinks, are forbid;" and the preference given to soup, even for breakfast, where the patient likes it: otherwise, cocoa, chocolate, and milk, are substituted. If his appetite requires it, calf's-foot jelly is given between breakfast and dinner, as often as the patient chooses; and, for this latter meal, he has any variety of animal food which agrees with him, but is obliged to confine himself to one kind.

Proceeding upon the opinion that nature is to be assisted in promoting the "resolution and discharge" of the tuberculous matter, he informs us that this is best effected by the compound galbanum pill, with sulphurated oxide of antimony, pulvis antimonialis, calomel, or cinnabar, with extract of conium and a small proportion of opium. To these are to be joined the use of the Iceland moss and sarsaparilla, or the seeds of the *phellandrium aquaticum.* This last remedy being almost, if not entirely, unknown in England, we shall take this opportunity of laying before our readers some particulars regarding its medical history, taken from the work before us. It was recommended by ERNSTING, in 1739, in the cure of ulcers, intermittents, and pectoral diseases; and again, in 1765, by LANGE, who bears very favourable testimony to its powers in the cure of consumption. Since that time, cases of recovery from phthisis under the use of this remedy have from time to time been published in the various periodical works of Germany. The seeds are the parts employed, and the dose is from a scruple to a drachm two or three times a-day. Our author has tried the phellandrium; and, when the pulse is quick, the cough frequent, and expectoration scanty, has uniformly found it to do harm; but, in the form of consumption described above, and in obstinate cases of chronic bronchitis, it has been of service.

In the treatment of pulmonary disease, the greatest attention is paid by our author to every circumstance connected with temperature. "When the weather is windy, raw, and cold, the patient ought to keep within doors. I do not permit those who cough much even to go into a passage, or into the staircase; or, if they quit their chamber, in order to have the air completely ventilated and renewed, (which ought to be done twice a-day,) the patient puts on some additional covering, in cold weather, before passing into the other apartment; which apartment ought to be immediately adjoining the one he leaves." (p. 95.)

In the various forms of phthisis, the cough is one of the symptoms to which the attention of the patient and his friends is most anxiously directed, and to alleviate which recourse is frequently had to stimulant expectorants,—as the squills, ammoniacum, &c. This practice is reprobated in the strongest terms by Sir Alexander, as having a tendency to excite inflammatory action in the lungs, and lead to the consequent formation of vomicæ. The remedies which he regards as most effectual in counteracting tubercular phthisis are the red sulphuret of mercury combined with antimony, of which the sulphuret seems to be a favourite preparation. Opium, hyoscyamus, and conium, are regarded as important adjuvants in allaying the irritation. On the subject of hydrocyanic acid, our author does not seem to have made up his mind, as he speaks of it differently in different passages: thus we are told, at page 102, "In the very advanced cases of tubercular phthisis, whether chronic or acute, the hydrocyanic acid appears to me, from repeated trials, to be quite inadmissible;" at page 111, "Digitalis and the hydrocyanic acid will, in general, do no harm in the very advanced stages of the disease;" and again, at page 115, hydrocyanic acid is mentioned, with other narcotics, as the "chief palliatives." Myrrh and galbanum, being less stimulant than ammoniacum, are of "considerable advantage," when combined with antimony, opium, &c. in cases where active inflammation does not exist, and the hectic fever is moderate.

The indications of cure in every case are said to be—1st, to prevent the formation of new tubercles; 2, to assist nature, either in causing them to be absorbed, or in the natural process of softening and discharging them; 3, to prevent the excess of action or inflammation; and, 4thly, to prevent the rapid loss of strength.

Among external remedies, blisters are first mentioned, and with the comment, that they are always safe, and generally of much use in relieving the pains in the chest which accompany the progress of tubercles. The same favourable testimony is given with regard to the pustular eruptions excited by tartarized antimony. Of issues, and the other forms of artificial ulceration, the author speaks less favourably; having sometimes seen them produce irritation to so great an extent as to render their discontinuance unavoidable.

The remedy, however, most efficacious in softening the tubercles, and effecting their removal, is said to be tar-vapour; but, as we have already selected, from various parts of the volume, such information on this subject as appeared to us most important, we must decline entering upon it again.

Emetics are said to do harm when tubercles are in their first stage, and the cough is consequently dry; they are likewise of doubtful efficacy in advanced stages of the disease, "as the vitality is sometimes greatly exhausted by their operation."

When, however, the expectoration is very copious, our author thinks they may prove beneficial under proper restrictions,—1st, by causing the fluids in the lungs to be quickly expectorated; 2dly, by stimulating the absorbents, by which, part of the fluid in the bronchiæ being removed, less remains to be coughed up; and, 3dly, by unloading the stomach when it is oppressed by its contents. Preference is given to the emetics which operate quickly without nausea.

Exercise is recommended, but with more attention to the state of the weather than is generally practised: the chief circumstances to be attended to are, to avoid exposure to cold, dust, and fatigue.

Steel is unequivocally condemned as a "most injurious medicine in tubercular phthisis;" the watery extract of myrrh is said to be an "excellent tonic;" and "no good" was derived from the cinchona. The colliquative sweats are most advantageously checked by cutting short the febrile paroxysm; and, to effect this, water fully charged with carbonic acid, with a little Sauterne or Chablis wine, may be given during the hot stage. In some cases, the patient may be spunged with cold vinegar and water in very hot weather.

Of diet we have already spoken. Some remarks on the complication of phthisis with syphilis, rheumatism, and hepatitis, conclude the chapter: when these occur, the remedies proper to counteract the more prominent evil are to be employed.

Our account of this part of the work, it will be observed, is strictly analytic; and, before entering upon another subject, we may be allowed to observe, that the result of our own experience of iron would have induced us to give a different report of its efficacy in the treatment of phthisis. This medicine, in some of its forms, particularly the sulphate, combined with conium or hyoscyamus, has appeared to us a very useful remedy in consumption, where, after the acute inflammatory stage has passed away, the cough and expectoration become very troublesome.

*Hemorrhagy from the lungs* is supposed to take place in three ways: first, it may arise from "fatal congestion," and produce suffocation by the quantity poured into the bronchiæ; secondly, the congestion may be relieved by the loss of blood, and the disease disappear for a longer or shorter period. "The third and most common way is that of inflammation," generally ending in ulceration. Original malformation of the chest, and the existence of tubercles in the lungs, are the chief exciting causes of hæmoptysis: when it arises from the latter, it is but one of the symptoms of phthisis; but, when caused by the former, it may, in the opinion of our author, be much relieved by the interference of art. M. AUTENREITH proposes that the patient should accustom himself to take inspirations, the hands being placed on some firm support, with a view of gradually expanding the lungs and enlarging the capacity of the thorax; a practice, the prudence and efficacy of which we are much inclined to doubt. It is proper to observe, however, that M. Autenreith himself cautions the patient against using this exercise to such an extent as to produce pain; and our author thinks "great moderation necessary in its employment." More confidence, Sir Alexander seems to think, is to be placed in throwing the arms back, so as to make the pectoral muscles act on the ribs, as in the exercise with dumb-bells. The necessity of attending to the attitudes of the patient, so as to prevent young persons from acquiring a habit of stooping, is also insisted upon: the horizontal position he regards as the most proper for habitual employment in reading. The tar-vapour is inadmissible in hæmoptysis. The general practice in such cases consists "in attempting to produce a diminished action of the heart and arteries, by cold externally applied, by bleeding, by cooling purges, and laxatives; and to arrest the hemorrhagy, by astringents, such as the infusion of roses, with sulphuric acid, alum, and, in some cases, the employment of superacetate of lead and astringent extracts, &c." The greater part of this treatment, the author immediately adds, is "irrational and hurtful." We confess that we were rather startled on reading this assertion, and had prepared to enter our protest against its accuracy; but our dissent was considerably qualified by finding, a little further on, that "a full bleeding is always useful;" that blisters are to be applied; and nitrate of potass, sulphate of magnesia, and cream of tartar, to be given internally, and the "head, neck, and chest left exposed to the cool air:" so that, in truth, the practice of our author differs less from that generally employed than he seems to be aware, or than the censure passed upon the means usually adopted would seem to indicate.

The principal circumstances insisted upon, in addition to those already mentioned, is keeping the feet, legs, and hands warm, either by woollen clothing or immersion in water of a proper temperature; and the condemnation of astringents, as sulphuric acid and sugar of lead. In this we cannot agree with the author, especially as regards the latter medicine, which we are in the daily habit of employing with much apparent benefit, and without any apparent evil, in the class of cases under consideration.

When the hemorrhagy returns in smaller quantity, remaining, as it frequently does, for many days or weeks, the inexpediency of repeated venesection is judiciously pointed out. In such cases, much good has sometimes appeared to result from small doses of the *pulvis ipecac. comp.* The following passage is worthy of attention:

"There are among the minutiæ of practice certain rules which appear so self-evident as to justify their being passed over in silence by systematic writers; and yet some of them are of importance, and do not always occur to a very young practitioner. Among these is the rule, in all hæmorrhagies from the lungs, especially active hæmorrhagies, to observe, as much as possible, an upright posture of the body, both by night and day. The patient ought to sleep in an high-backed armed chair, with his feet extended when he grows tired of letting them hang down. The observing the greatest possible silence is also another essential rule. The patient ought not to speak: he ought to endeavour, as much as is in his power, to subdue the cough by his will, or resist the habit of coughing from every slight irritation about the fauces or trachea. In active hæmoptoe, every thing the patient eats and drinks ought to be cold; and scarcely any animal food ought to be allowed until convalescent, and even then sparingly. In this respect, as well as in the principal medicines, the treatment of this complaint differs essentially from that of tubercular phthisis."

In treating of the form of consumption which arises from *neglected peripneumony*, the author takes occasion to state his dissent from the opinion of LAENNEC with respect to the infrequency of its occurrence. The remedies recommended after the inflammatory stage has passed away, are the inhalation of tar-vapour, at first in a very dilute state, emetics, issues, and mild tonics,—as the decoction of *genuine* Iceland moss, watery extract of myrrh, sulphate of zinc, and superacetate of lead.

*Bronchitis*, when it passes into the chronic form, and is accompanied with fever and expectoration resembling pus, constitutes a species of consumption,—the *phthisis pituitosa* of the German writers. It is in this variety of the disease that there is most evidence in favour of the efficacy of tar-vapour, according to the experiments of HUFELAND and NEUMANN, and Dr. FORBES, to whose paper we have already referred.

The subject of bronchitis leads the author to speak of one of the exanthemata from which it frequently arises,—viz. measles. In a chapter upon this subject, the free admission of cool air, and the exhibition of cold drinks, as practised in other similar complaints with so much benefit, are condemned, as liable to produce "irreparable injury." In place of this, we are advised to prohibit all drinks except those which are tepid, and preserve, with the utmost care, "a well-regulated temperature." In a severe case, "the salvation of the patient depends almost entirely on the due employment of general or local blood-letting, blisters, and, above all, the avoiding, with the greatest possible care, the slightest impression of cold air, *let his apparent uneasiness and restlessness from heat be what it may*."

On this subject we shall only remark, that we dissent entirely from the author, being constantly in the habit of employing the cautious use of cool air and cold drinks, without seeing any of those injurious consequences alluded to by the author. Neither, indeed, do we think that any sufficient arguments are advanced to warrant a method of treatment in opposition to the analogy of other exanthematous diseases, and which would appear, by the concluding part of the above quotation, which we have put in italics, to produce, in some instances, those very symptoms which we are anxious to guard against.

A short chapter on *laryngeal and tracheal consumption*, gives a sketch of this form of phthisis, which, although not very infrequent, is, we believe, often overlooked, and has not received the attention it deserves. We can bear testimony to the accuracy of the following description:

"The ulceration of the cartilages of the larynx, when once it begins, produces, in a very short time, a most remarkable effect on the general health of the patient. He loses strength and flesh more quickly than in most diseases, and the sound of his voice is immediately changed in tone. The patient cannot cough out, as it were, and the sound produced by the act of coughing is singularly husky. If a pressure be made on each side of the trachea, while the patient is made to speak, a cough, with a very peculiar stridulous sound, is generally excited; and this may be considered as a true test, or as a pathognomonic symptom. In bad cases, a pain extends to the throat and eustachian tube; but, on inspecting the throat, it is seldom that we discover much inflammation. The cough is frequently excited, and continues in one uninterrupted paroxysm until a quantity of frothy mucus, mixed with minute portions of purulent matter, is extricated. Hectic fever, night sweats, and colliquative diarrhœa, occur as the disease proceeds."

The vapour of tar "promises to be effectual in most cases, if taken in time:" besides which, an uniform temperature is enjoined; the patient is to speak as little as possible, and to take Iceland moss and the balsams of copaiba and tolu, with sulphur. "Opium and its preparations do harm." We have never tried tar-vapour, nor the other remedies here mentioned, with the exception of opium, and from this we have seen the greatest relief to the sufferings of the patient: indeed, the difficulty of swallowing which is frequent in this complaint,—the harrassing cough, and spasmodic action sometimes brought on about the throat, have appeared to us to indicate narcotics more decidedly than any other form of consumption. It is to be observed, we speak of ulceration of the cartilages of the larynx.

The question,—is consumption hereditary? is next discussed, and decided in the affirmative; an opinion from which few of our readers, we imagine, will be inclined to dissent. The plan of intermarrying the tainted with the healthy, in order to ameliorate the breed, is condemned in the strongest terms. Those interested in the matter will find the opposite doctrine systematically advocated by M. PETIT, in the Dictionnaire des Sciences Medicales.*

The only part of this work which it remains for us to notice, is the discussion of another question,—Is consumption contagious? This likewise is decided in the affirmative; the result of Sir Alexander's experience being, "that, when the air of an apartment is strongly impregnated with the breath of any one, or any number of consumptive people, labouring under the advanced stages of any variety whatever of purulent phthisis, a poison is often produced by the chemical influence of temperature on a confined air, thus loaded with vitiated animal effluvia; just as the poison of scarlatina is often generated from temperature acting on air loaded with the breath and perspirative matter of a number of children, when confined in small apartments." The author informs us, that by far the greater number of examples of this contagion occurred while he was physician to a Dispensary in London:—it is remarkable that we, being placed in a situation exactly similar, have never met with any instance of the kind. The question is one of those which would admit of much discussion, ending, like many other discussions, in very unsatisfactory conclusions. This, however, seems clear, that, as many respectable writers,† besides the one before us, regard phthisis as contagious under certain circumstances, so it is most prudent to prevent the healthy from unnecessary exposure to the source of contagion, whether real or imaginary.

---

ART. II.—*History and Method of Cure of the various Species of Epilepsy: being the second Part of the second Volume of a Treatise on Nervous Diseases.* By JOHN COOKE, M.D. F.R.S. F.A.S. Fellow of the Royal College of Physicians, and late Physician to the London Hospital.—pp. 235. Longman and Co. London, 1823.

THE work at the head of this article completes the "Treatise on Nervous Diseases." The design of the distinguished author, as set forth in his preface, was to give a clear and comprehensive view of all that is important and useful to be known on the subject of which he treats, collected from the best authorities, and methodically arranged; and although, in observing the unaffected and perspicuous manner in which this has been executed, we may be disposed to regret that Dr. COOKE has not gratified us with more copious details of his own opinions and experience, we are compelled to acknowledge that such would not have been in keeping with the plan which he had laid down. At all events, it must be regarded as a very unusual subject of

---

* Tome xxi. art. Héréditaire.

† Galen, Lommius, Reverius, Morton, Hoffman, Van Swieten, Selle, Fritze, Wiclmann, Vogel, Emale, Baume, Rush, Darwin, &c. &c.

regret, that the author of a modern medical work has spoken too little of himself.

Before we enter upon our analysis, we would say one word of the design. It cannot have escaped the observation of any one acquainted with medical writings, that many works issue from the press, announcing *new* theories, *new* remedies, or giving *new* views of the subject of which they treat, without having any other claim to novelty than that of being *new* to their author; and, in other instances, although his book may contain much that is new and much that is true, it sometimes turns out that the true is not new, and the new is not true. Now, this inconvenience would be entirely obviated, if medical men qualified for the task, as in the example before us, would take up particular subjects, read and digest what has been recorded, and draw up such an account as would guide future inquirers, —preventing the unnecessary repetition of experiments,—and saving the trouble of inventing new theories in opposition to old facts. Such we conceive to have been the design of Dr. Cooke; and, had he fellow-labourers enough, something more nearly approaching to a complete system of medicine might be obtained than any that has yet appeared. Such a work would require many to accomplish it: no one individual can reasonably hope to do more than a part.

*Definition and History*.—ARETÆUS is the only writer among the ancients who has given a satisfactory account of this disease, or one which can be consulted with any considerable advantage, although mention is made of it at some length by HIPPOCRATES, GALEN, ÆTIUS, and others. The names assigned to it have been various, as *morbus sacer*, *comitialis*, *Herculeus*, *caducus*, &c.; appellations derived from real or imaginary circumstances connected with the symptoms. *Epilepsy*, however, is the term now generally preferred, and designates a disease which, in the words of our author, consists of "paroxysms of convulsion returning at uncertain periods, accompanied by an abolition of sense and voluntary motion, and ending in somnolency or complete sleep." The invasion of a paroxysm is sometimes sudden, and in other instances preceded by certain premonitory symptoms, some of which,—as pain or giddiness of the head, various affections of sight and hearing, languor, drowsiness, or torpor, —are common to this disease and apoplexy. Others, however, are peculiar to epilepsy; among which the most remarkable is a sensation which usually commences at the extremities, and passes towards the head, called *aura epileptica*. This symptom occasionally conveys the idea of insects creeping along the skin, and is then called *formicatio*.

"This symptom, or, as some call it, cause of epilepsy, has been noticed by almost all writers on the disease, both ancient and modern,

"Galen says, that in the early part of his life he had seen an epileptic boy, about thirteen years of age, who spoke of this affection as a certain something, which he could not clearly describe, ascending along the thighs, the loins, the sides, and the neck, up to the head; which was followed by a total loss of memory or recollection. He also mentions a case in which the sensation was compared to that of a cold wind ascending to the head. Paulus Ægineta likewise describes this *aura frigida* as going from the legs, or from the fingers, to the head. He speaks of a woman who during pregnancy had epileptic fits, which were preceded by a sense of a cold air ascending from the uterus to the brain. Schenckius has described this sensation as it affected a boy of twelve years of age, who was subject to epilepsy."

Various attempts have been made to explain this phenomenon, but without much success, and the general opinion among modern writers is, that it proceeds from some irritation of the nerves communicated by their medium to the brain. It was remarked, however, by CULLEN, that the *aura epileptica* seldom follows the course of individual nerves, but generally passes along the integuments. It appears, from the testimony of various authors, that this symptom occasionally arises from causes strictly local, and ceases on the removal of such local irritation.

The ordinary symptoms of epilepsy are well described, but our limits confine us to what is remarkable. SCHENCK gives an example of the disease, which was ushered in by "a strange involuntary whirling of the body, round and round;" BOERHAAVE one in which the lips were "alternately contracted and elongated, thrust out into a sharp beak, and then drawn back with such celerity as to make its beholders giddy;" VAN SWIETEN speaks of the forehead and scalp being convulsed, so as to make the hair stand on end; and TISSOT has seen ecchymosis about the forehead and eyes, while the rest of the face exhibited here and there small red spots, which disappeared after a short time.

"In the paroxysm, the symptoms above mentioned, sometimes sooner, sometimes later, gradually give way. The convulsions become less violent; the breathing more free; the pulse more full, slow, and regular; the countenance more composed; and the patient falls into a state of somnolency or profound sleep; on awaking from which, he by degrees returns to his usual state, nothing more than languor or lassitude remaining.

"The paroxysms of epilepsy in different cases greatly vary, both as to their degree, their duration, and the time of their return. When the disorder appears in its worst form and in its highest degree, it is attended with the frightful circumstances and symptoms above mentioned; and the description which I have given is by no means an exaggerated account of it. Much more frequently, however, it appears in a milder form, when the patient, with or without warning, falls to the ground, remains for a short time convulsed, and soon passes into a state of quiet sleep. Sometimes the fit happens in the night, and the patient, on awaking in the morning, feels nothing more than a slight weakness and oppression. In some cases, the epileptic person does not fall to the ground, and experiences no inconvenience further than that of some agitation of the head or limbs; attended, however, with a loss of sensation and voluntary motion, in a greater or less degree. In many cases, even of slight epilepsy, some foaming at the mouth is observable. —Both in the severe and the mild disease, the patient, on recovering, seems wholly unconscious of what has happened during the paroxysm, only recollecting circumstances which occurred previous to its accession. Aretæus has described epilepsy in both its forms, and has given it a place both among the acute and chronic diseases. Speaking of the acute epilepsy, he says, it is an evil of a various and portentous kind, fierce in its paroxysms, acute and pernicious, or deadly; for sometimes a single fit proves fatal."

*Distinctions and Causes.*—Epilepsy is considered under the two divisions, of idiopathic and symptomatic; the former being a primary affection of the brain, and the latter affecting that organ by secondary influence. Some pathologists are of opinion that predisposition to the disease is not necessary, and that a " new and strong impression" may excite epilepsy, even where there is no peculiarity or imperfection in the organization of the brain or nervous system. Our author, on the other hand, thinks it evident that a certain state of the brain, existing in particular individuals, renders them much more liable to the disease than others differently constituted: and, indeed, it must be obvious to all, whether a predisposition be always present or not, " that the disease is excited in some persons by causes which do not produce it in others." The hereditary nature of this tendency to the disease is strikingly illustrated by an example from Tissot, who mentions the case of a father, eight sons, and three grandsons, who were all affected with it. In other instances, the disease has been known to lie dormant for one or more generations, and then re-appear with great force. It does not seem to be clearly ascertained whether epilepsy be more prevalent among men or women, but the experience of our author favours the opinion of the male sex being the more obnoxious to it. Although the continuance of the disease has a tendency to weaken the mental powers, and even frequently to lead the way to idiocy, yet its occurrence does not appear incompatible with mental acquirements of the highest order: witness, Julius Cæsar, Mahomet, Petrarch, and Rousseau. When the predisposition exists, it is more readily called into action by a plethoric state of the body; and, when the disease has already occurred, habit becomes a very powerful cause in leading to its recurrence.

Among the exciting causes which act primarily upon the brain, are enumerated many purely mechanical, as malformation, tumors, &c.; and the question, why the same cause should sometimes produce apoplexy, and at others epilepsy? is answered by reference to the explanation of M. PORTAL. "He has proved by experiments, that pressure on the brain in a great degree will occasion apoplexy, and in a smaller degree convulsions: thus apoplexy, by a diminution of the power of the exciting cause, may end in epilepsy; and epilepsy, by an increase of it, may terminate in apoplexy: and this, indeed, we very often find to be the case. Perhaps the different symptoms of these two diseases arising from compression within the cranium, may in some measure depend upon the particular parts compressed."

The influence of the passions are well known, and of these fear seems to be the most powerful.

"Van Swieten informs us, that a lady of a strong constitution, who had always enjoyed robust health, was, during pregnancy, so much terrified by the appearance of a dreadful fire in the neighbourhood, as to be affected by epileptic fits, which at length proved fatal.—I never saw, says an eminent French physician, a more distressing case of epilepsy than that of a female, who, on receiving a very gross insult from an insolent blockhead, was two hours afterwards seized with a violent attack of that disease, which returned three times in the following night; and, although she had the best advice, the disorder increased, from which she was never free for more than a day, for many years, dragging on a most miserable existence.*—A celebrated German physician informs us, that in six out of fourteen epileptic patients, under his care in the hospital of St. Mark, at Vienna, the disease had been occasioned by terror.†—A monk at Rome, on suddenly receiving the news of the death of his brother, became epileptic, and suffered two or three fits daily.‡—A man travelling by night met a large dog in a narrow path, and fancying that he was seized by the animal, he arrived at home in great terror, and the next morning had a violent fit of epilepsy, which afterwards returned a great many times. It always began with a violent cramp in the hands, which, ascending by the throat, and then descending to the heart, deprived him of sense.§—A young man, having witnessed some of the dreadful events at Paris on the horrible 10th of August, became affected immediately with this disorder.||—We have several cases on record in which epilepsy appears to have been produced by the power of imagination. A boy, of a sound and good constitution, became epileptic immediately after drinking out of a cup from which he had seen an epileptic person drink.¶—A robust man having dreamed that he was pursued by a bull, on awakening in great agitation and in a state of delirium, in a quarter of an hour fell down in a strong fit.**—A servant maid at Leipsic, endeavouring to untie some knots, and fancying

* Tissot. † Locker. ‡ Schenck. § Tissot.

|| Maisonneuve *Recherches sur l'Epilepsie.*

¶ Schenck. ** Tissot.

that one of them was made by a sorceress, became so terrified that she was seized with this disorder, which was followed by several others.*

*Dissections*.—The appearances which have generally presented themselves on the post-mortem examinations of persons subject to epilepsy, are such as we are led by analogy to regard as capable of producing irritation of the brain. These are simple vascular congestion, or the existence of effusion, abscess, tumor, or spiculæ of bone, within its substance. But the most important observations on this subject are those by M. Wenzel, the ingenious professor of anatomy and physiology at Mayence, who instituted a series of examinations for the express purpose of determining the morbid derangements which give rise to epilepsy. A society was formed to facilitate this investigation, consisting of various eminent individuals, who adopted different modes of treatment; and, when these proved unavailing, the task of examining the body fell on M. Wenzel, who was so anxious to determine the appearances of the brain peculiar to epileptics, that he always carried with him, on these occasions, the brain of some one who had never suffered from that disease. The results of twenty such examinations are given, in all of which the cerebellum was remarkably diseased; while in fifteen the brain proper was perfectly sound. "In ten instances, the pineal gland was almost entirely of a grey colour;" it was always softer; and in every instance, except two, it was smaller than natural:" in those two, however, it was much enlarged. The cerebellum was morbidly affected: "with respect to its surface, its consistence, its colour, its size, and the state of its internal parts." The colour of the cerebellum was sometimes paler than natural, but more generally "of a dusky red of different shades, and approaching to blackness." In ten instances, a yellow, friable, solid matter was found between the lobes, producing a separation and loss of substance; sometimes a viscid, semi-fluid lymph was found separating the lobes; "sometimes, upon the superior surface of the cerebellum, spots were produced by a collection of perfectly white, or yellowish brown, lymph, which had become solid." Dr. Cooke justly remarks, that when we consider that M. Wenzel found the cerebellum diseased in every epileptic patient, without exception, we cannot but feel surprised that these morbid changes should have escaped the notice of all preceding anatomists. Besides these appearances peculiar to the head, various others have been described as affecting the thoracic and abdominal viscera. Dr. Prout (of Paris) found collections of worms in the intestines; but in other instances, according to our author, "no marks of disease whatever could be found within the

* Tissot.

cranium, the thorax, the abdomen, or in any other part of the body."

Powerful impressions made on any of the senses may give rise to a fit of epilepsy; and it is mentioned by ARETÆUS, that the ancients, when they purchased slaves, were in the habit of exposing them to the unpleasant fumes of the lapis gagates (jet), in order to find out whether they were subject to this complaint. In other examples, particular sounds produce the same effect; and, we believe, Mozart afforded an illustration, being thrown into convulsions, when a boy, by the sound of a trumpet.

Among the causes acting secondarily upon the brain, irritation of the stomach and bowels is probably the most frequent. A curious example of this is given by M. MAISONNEUVE, in the history of eighteen persons, who, having suffered shipwreck, were obliged to live upon indigestible food, and to drink sea-water during several days: the whole were affected with epilepsy, and only four recovered from the disease. Further illustrations of this are to be found in the work of Dr. PROUT already mentioned; and our author says, "I have seen some cases of epilepsy, in which the disease has ceased on the discharge of worms; and others in which it appeared to have been cured by anthelminthics, although no worms could be discovered in the evacuations." Various other sources of irritation are mentioned, originating in the hepatic, urinary, and uterine systems; but upon them our limits forbid us to enter.

Some observations are made on the connexion between epilepsy and various other diseases, as apoplexy, chorea, hysteria, &c.; and the chapter concludes with a few remarks on the proximate cause of the disease. How little our author is satisfied with the result of this inquiry, appears from the following words: "Notwithstanding the pains which many ingenious physiologists have taken in endeavouring to understand this subject, it still remains very obscure; and perhaps we must, in candour, admit the truth of what has been said by the learned author of a dissertation on Epilepsy, who, speaking of its proximate cause, observes, "Est quasi terra incognita in qua quisque pro voluntate sua vagatur, et viam diligit jam factam aut facit. Auctores de hac re multas plausibiles et populares fabulas effinxerunt hoc vero omnia novimus esse nihil."

*Diagnosis and Prognosis.*—Hysteria is probably the only disease with which epilepsy is liable to be confounded; and even here our author is of opinion that it may be distinguished by the foaming at the mouth, and other symptoms mentioned in the history of the disease, together with the absence of those symptoms regarded as characteristic of hysteria. We are, however, inclined to believe the diagnosis between these two diseases to be often extremely difficult; or, rather, that they sometimes run into each other, making distinction impossible. We lately saw a delicate female, under twenty years of age, who, at the period of quickening, was subject to fits, accompanied with complete insensibility and congestion in the head, and convulsions of the limbs so violent as to require several powerful men to prevent her from hurting herself, and having in all other respects the appearance of violent epilepsy. As the fit passed off, the patient was seized with involuntary laughing and weeping alternately, giving the disease the character of hysteria.

The prognosis, except under particular circumstances, must be regarded as unfavourable. When the disease is caused by any thing acting permanently in the brain, a cure can scarcely be looked for; but when it arises in early life, particularly before puberty, the natural changes in the constitution frequently give rise to its spontaneous disappearance.

The *treatment of epilepsy* is considered in the fifth and last chapter, which is likewise the longest and most important. Where vertigo and other symptoms of plethora about the head make their appearance in persons of full habit, no question can exist of the propriety of speedy depletion, by blood-letting and purging; but our author cautions us against the use of too free evacuations in advanced age or debilitated habits. Various means have been recommended with a view of preventing the accession of a paroxysm, when its approach can be seen. Some have administered emetics; but their use is condemned by Dr. Cooke, as he fears the fit might rather be promoted than obviated by such practice. One of the most common, and probably the most effectual, means of arresting a fit, in those cases in which the aura epileptica occurs, is the application of a ligature between the brain and the part whence it proceeds. During the paroxysms, the following treatment is recommended:

"The patient should, as soon as possible, be placed in a bed or sofa: the head should be somewhat raised, and such parts of the dress removed as may press upon the vessels of the neck. The convulsive action, when violent, should be restrained; and every precaution should be taken, to prevent injury to the patient and those around him.—It often happens that the tongue is lacerated by the spasmodic action of the muscles of the jaws: in order to guard against this mischief, a piece of cork, or other soft wood, or a napkin or handkerchief properly rolled up, should be introduced between the teeth.—Some persons are of opinion that, when the hands are powerfully clenched, the thumb and fingers should be forcibly straightened; but I see no probability of advantage from such a practice: on the contrary, if much force were to be employed, I think it might be prejudicial.

"In addition to these means, some physicians, especially in former times, have recommended a variety of external applications and internal medicines, with a view of shortening the paroxysm, and mitigating the violence of its symptoms.

"Some have advised bleeding in the fit, on the supposition that the brain is overloaded with blood, and that, by lessening partial plethora, relief might be obtained. In a case of epilepsy arising from a suppression of the menses, we are told that Hoffman produced much mitigation of the symptoms by bleeding in the foot during the paroxysm; but that this treatment did not prevent a return of the fit on the following day. To say nothing, however, of the great inconvenience of the abstraction of blood under strong general convulsive muscular action, I doubt whether any mitigation of symptoms would, in general, be thus obtained; more especially as we are informed, from high authority,* that spontaneous bleeding from the nose has occurred in attacks of this kind, without apparent relief. Bleeding during the paroxysm is calculated, says an accurate observer, to lessen the strength of the patient, but not the power of the disease.†

"After attention to a proper position of the body, to the removal of ligatures, and to the means of preventing injury to the patient, or others, by the involuntary convulsive muscular actions, every thing further during the fit, such as the application of acrid or volatile stimulants to the mouth or nose, frictions, &c. are, I believe, useless, if not dangerous. In many, indeed in the generality of cases, the symptoms of this disorder are mild, and scarcely any thing need be done to mitigate them. On recovery from the fits, if the patient be disposed to sleep, it may be encouraged; and if, when he awakes, he complains of languor or faintness, mild cordials may be administered."

The effect of restraining the flow of blood to the head is curiously illustrated by a case which occurred to Mr. Earle; being however, as Dr. Cooke justly remarks, one of an apoplectic rather than an epileptic nature. A young gentleman received a severe fall, in consequence of which he became subject to fits, one of which is thus described:—"I found him perfectly insensible, with stertorous breathing, and his eyes and whole countenance evincing great determination to the head. He had passed his urine and fæces involuntarily. I immediately bled him from the arm and jugular vein; but he did not evince any signs of returning sensibility until I had taken between forty and fifty ounces of blood." Observing the powerful action of the carotid, Mr. Earle was led to try the effects of compression; and the result was, that the duration of the fit was much diminished. The nature of the experiment was explained to himself and his friends, who were thus enabled to put it to the test, by applying pressure on the occurrence of certain premonitory symptoms, by which means the attack was very frequently effectually arrested in its progress. At one time it

* Tissot. † Heberden.

became matter of serious consideration with Mr. Earle, whether it might not be admissible to tie one or both carotids, as the blood-vessels of the head, passing through bony canals, cannot become dilated by the obstruction of the corresponding artery, with the same facility as in other parts.

A powerful mean of obviating increased vascularity in the head is afforded by the application of setons and issues; many examples of the efficacy of which are to be found in practical writers, and they are recommended by our author in the kind of epilepsy at present under consideration. Purgatives are also very favourably spoken of, especially by HAMILTON, PRICHARD, and ABERCROMBIE.

When, however, the disease depends upon debility, a different plan of treatment must be adopted; and for this purpose a variety of tonic and antispasmodic remedies have been recommended. Among these, the cold bath is much praised by TISSOT; but Dr. Cooke informs us, that he would not venture to recommend it, if there was any perceptible fulness of the vessels about the head. Others, probably in reference to the well-known cure effected by BOERHAAVE, in the Orphan House at Haarlem, have advocated the excitement of a certain degree of fear; and, in the eighteenth volume of this Journal, is an account of a lady who was cured of epilepsy by a sudden fright. Such means, however, our author justly remarks, are of very doubtful utility, and too little under control to be consistent with safety.

Among the vegetable bitters, the misletoe obtained the greatest celebrity, but, with its companions, has now fallen into disrepute, and the mineral tonics have of late years taken the lead; but the very fact of *silver*, *zinc*, *copper*, *lead*, *arsenic*, and *mercury*, having all found advocates, sufficiently shows that none possesses such a decided superiority as could be wished. Dr. POWEL was the first to show the great extent to which the nitrate of silver may be given with impunity, if not with advantage. The result of his experiments was, that three times as much can be administered in the form of pill than in solution; fifteen grains of the solid caustic being borne by the stomach as readily as five grains in solution. The efficacy of this medicine is further supported by the testimonies of Dr. BAILLIE, Dr. RICHARD HARRISON, Dr. ROGET, and Dr. JAMES JOHNSON; and it is remarkable that, although several physicians have seen no benefit from its administration, yet none mention any ill effects as resulting, with the exception of M. GEORGET, who seems to have condemned it without sufficient grounds. But, although no dangerous symptoms seem to result from the nitrate of silver, when administered with due caution, still the discoloration of the skin which sometimes follows, must be regarded as one of serious inconvenience. The exact nature of this phenomenon does not appear to be ascertained; for, although the dark colour generally remains permanent, yet, in a case mentioned by Dr. ALBERS, of Bremen, the blueness diminished, or even almost entirely disappeared, when the patient held her arms in an erect posture. Another very singular fact has been related by Dr. ROGET,—viz. the occasional appearance of the discoloration so long as six months after the exhibition of the medicine had been discontinued. In another case, mentioned by Dr. VETCH, "a lady under his care, after a long-continued use of this remedy, became discoloured in the upper part of the body, whilst the colour of the lower part was unaltered; and, in both eyes, the iris, which was naturally of a black or deep brown, was changed to a light blue."

*Zinc* probably comes next in reputation to silver, and has been recommended by GAUBIUS, HART, GUTHRIE, FOUQUET, PERCIVAL, and RUSH. Among these, Dr. Guthrie seems to have pushed the use of the flowers of zinc to the greatest extent; having in one case exhibited eight grains the first day, and gradually increased the dose during a month, till it amounted to two scruples, when the disease had disappeared. Our author, however, states that his own experience is not in favour of this remedy in epilepsy, although he has found it useful in chorea and other nervous affections. He likewise suggests, that the different accounts of the effects of zinc may, perhaps, be reconciled by the fact of the *cadmium* which it contains varying in proportion; which ingredient may possibly be the active part of the composition.

The *cuprum ammoniatum* is another remedy of some celebrity, which was introduced by Dr. CULLEN at Edinburgh, where it has been principally employed; and some striking cases in which it has proved beneficial are to be found on record. At the same time, it must be acknowledged to be not less uncertain than similar remedies, having been given in doses of four grains without avail.

The *acetate of lead* has been recommended by Dr. RUSH, in doses of two grains three times a-day; and Dr. SPENCE, another American physician, took it, in his own case, to the extent of eight grains twice a-day for three or four weeks, with apparent advantage.

*Tin* was extensively used by Dr. FOTHERGILL; and, more recently, Dr. SHEARMAN* informs us that the *elutriated oxyd of tin*, in doses of from two scruples to a drachm night and morning, for about four days, and then alternated with a purgative, has appeared to him to have considerable power over this disease.

* *London Medical Repository*, vol. xviii.

*Arsenic* has likewise found its advocates; and we have ourselves employed this medicine in three cases of epilepsy, with apparent benefit: one patient, who had been subject to a fit every fortnight or three weeks, remained free from any attack for eighteen months after a course of the liquor arsenicalis employed for several weeks.

*Phosphorus* is thus spoken of:—

"In the Journal of Dr. Hufeland, professor of medicine at Jena, we have an account of the good effects of phosphorus in epilepsy. The use of this remedy was discovered by accident. A young lady, subject to violent spasms of the stomach and bowels, terminating in fainting or an epileptic fit, took about an ounce of water, containing two drachms of phosphorus, instead of an infusion of peppermint, on the accession of certain symptoms, which by experience she knew to be the forerunners of an epileptic paroxysm. The consequence was, that the fit was completely prevented. Her physician, taking advantage of this accident, afterwards prescribed for her a mixture, containing six grains of phosphorus, half an ounce of oil of hyosciamus, and two ounces of peppermint-water. Of this mixture the patient took a table-spoonful every two hours, for two months, and became entirely free from the disease. The same physician prescribed phosphorus in three other cases of epilepsy, with success; but in some instances he found it hurtful. Dr. Hufeland considers phosphorus to be a dangerous remedy. He has known several instances in which, having been boldly prescribed by quacks, it produced much mischief. It cannot be given, he says, in a dose of more than two grains with safety. He found that larger doses always produced burning pains, and one grain was generally sufficient. With regard to form, he observes that it must be completely dissolved and involved, so as to prevent its stimulating the stomach too much. Well triturated with mucilage of gum Arabic, in the proportion of two grains to six ounces of water and an ounce of syrup, he obtained an active and pleasant emulsion, to which he added thirty drops of the anodyne liquor of Hoffman, and in this form he employed it without inconvenience."*

The *oil of turpentine* appears to be entitled to some confidence in certain cases of epilepsy, particularly those in which the irritation is kept up by worms in the intestines, or in which the menstruation is imperfect. Dr. LATHAM, Dr. YOUNG, Dr. PERCIVAL, and Dr. LITHGOW, have severally mentioned cases of the disease successfully treated with this medicine.

*Opium* seems not to have been used so extensively as might have been supposed, considering its power in repressing other forms of spasm. *Stramonium*, *digitalis*, and *hyoscyamus*, have all been recommended; but without any satisfactory evidence in their favour.

All the above remedies act by removing the predisposition to

* *Annals of Medicine*, vol. iv. p. 276.

the disease. Another indication is to remove or diminish the influence of the exciting causes; and on this subject our author enters pretty fully into the history of the actual cautery, and some of the other forms of escharotics and issues. Into these details it is not our intention to follow him, having expressed ourselves very fully on this subject in a recent Number.*

In epilepsy from enteric irritation, or depending on the state of the uterus, various purgatives are recommended, among which the oleum terebinthinæ deserves a trial: but for the particulars of their selection and form of administration, we must refer to the work before us, which, we believe, contains all on the subject of epilepsy which it is of importance to know.

We conclude in the words of the learned author:—" I have now finished what I had to communicate respecting the history, causes, and method of cure, of apoplexy, palsy, and epilepsy. In my account of these very important nervous diseases, I have endeavoured to abstract, to condense, to methodize, and to convey in clear and plain language, the best information I could collect from a great number of writers, both ancient and modern. I cannot flatter myself that, by the investigation which I have made of these obscure disorders, I have done much towards the illustration of their nature; but I do hope that the description I have given of the experiments, observations, opinions, and practice, of the most celebrated physicians in various ages respecting them, will prove, in some degree, useful, both by lessening the labours of the student, and by affording practical assistance to persons who are actually engaged in the duties of the profession."

---

### DIVISION II.

### FOREIGN.

ART. III.—*Lettere del* Professore VACCA BERLINGHIERI *al* Professore ANTONIO SCARPA, *sulla Legatura delle grosse Arterie e degli Arti.—(Letters of* Professor VACCA *to* Professor SCARPA, *on the Ligature of the large Arteries, and those of the Limbs.)*—pp. 32. Pisa, 1820.

ALTHOUGH surgeons in this country are pretty well agreed as to the best method of applying a ligature to the principal arterial trunks in cases of aneurism, yet, on the continent of Europe generally, and especially in Italy, many questions have been raised as to this operation, and warm contests have ensued, and still continue to divide the opinions of some of the most celebrated men, both in that country and in France. The shape,

* See our Review of Mr. DUNGLISON's translation of LARREY on Moxa.

and materials of which the ligature should be composed,—the mode of applying it immediately, or with the intervention of some substance between it and the artery,—the degree of force to be used in tying it,—and the propriety of removing it after the lapse of a few days, or of suffering it to fall off spontaneously,—have all been separately discussed; and each party has not only supported its opinions by arguments, but has also appealed to experiments, which have only served still further to increase the obscurity, and to render the decision of the question still more difficult. The letters which stand at the head of this article appear to us to have great merit, and to decide this point against the method proposed by Scarpa. At the same time, they may be quoted as models of professional controversy, being entirely free from all personalities,—from every vestige of ill-humour, jealousy, or rancour,—and establishing their claims to notice upon the solid foundation of observation and argument. As compositions, they are, however, far from faultless:—the matter is not very perspicuously arranged; in consequence of which, repetitions are frequent, and the argument is rather obscured by a redundance of expression.

We shall give a copious analysis of these letters; conceiving that, by so doing, we shall set this practical question in a clearer point of view than by any reasonings of our own; merely premising a few words relative to the employment of the ligature in this country and in France, showing the principles upon which this operation is founded, and the changes introduced by the celebrated Scarpa;—this will at once bring us to the pamphlet before us, the point from whence we started.

Although the ligature has now been generally employed as a mode of restraining hemorrhage for upwards of two centuries, it does not appear that the principle upon which this practice is founded was at all understood until these few years past, and that, principally, in consequence of experiments made in this country. In the course of the eighteenth century, various theories obtained credit with the profession: some authors attributed the closure of the vessel to the formation of a coagulum only; others considered that process to be much assisted by the contraction of the artery itself; and a third party ascribed the whole effect to the swelling produced in the cellular membrane; and so little faith seems to have been given to the efficacy of either, or both of these processes united, that other means were eagerly sought for to obviate the recurrence of secondary bleedings, which were frequent, in consequence of the real process of nature in closing the arterial trunks not being properly understood. The twisted suture, in consequence, found admirers and encomiasts; and, though experiments both on man and animals really proved the safety and efficacy of the plan, it has happily given way to an operation much milder, and certainly not less effectual. It would be a useless waste of time to revert to all the schemes that have at different periods been brought before the public to procure the obliteration of arteries, in cases of aneurism especially. To Dr. Hunter the world is indebted, most assuredly, for the great improvement in these operations; and, though his first attempts were (comparatively speaking) rude, he has the merit of having pointed out the right road, and was, in reality, the great and efficient cause of all the improvements that have subsequently taken place, and which now render the tying, even of the largest arterial branches, an easy, and we may say an every-day, occurrence. It may even be said, without much fear of contradiction, that the inconveniences which were found to attend his operation, were, in fact, the original causes of the inquiries that were subsequently instituted into the processes which nature employs for the obliteration of the artery, and which have consequently led to the adoption of the only safe practice in all cases,—that is, a practice founded upon principle.

The writings of Dr. Jones, Mr. Hodgson, and Mr. Travers, cannot, in this point of view, be too highly appreciated: the experiments which they instituted, and the conclusions deduced from those experiments, appear to us entirely satisfactory; and they have been confirmed by Beclard and others, who have repeated them with the same results. It cannot be necessary for us to detail the substance of the above experiments, because we hope and believe that no professional man of the present day can be ignorant of the main facts which those valuable works contain;—it only remains, therefore, to mention the peculiar view that Scarpa has taken of the subject,—in which he is joined by many of the Italian professors; and, in one point of his practice, (that of placing a compress between the ligature and artery,) even by Vacca himself.

Scarpa then, in the first place, uses a broad and flat ligature. He conceives that secondary hemorrhages arise from the protracted stay of the ligature upon the artery, producing, by its presence, suppuration and ulceration of its coats; and he therefore removes the ligature on the fourth day. This danger he considers to be farther increased by tying the vessel in the manner recommended in England,—that is, with the intention of dividing the internal and middle coats of the artery; and, lastly, he interposes a small compress of linen between the artery and the ligature, for the purpose of making the pressure as light as possible,—his object being merely to bring the sides of the vessel in contact with each other. His reasonings in support of these peculiar opinions we shall not combat ourselves, conceiving that they will appear in the course of our analysis of

M. Vacca's letters, and trusting that their refutation will be as apparent and satisfactory to our readers as to ourselves.

Our author, after accounting for the delay that occurred between the receipt of Scarpa's letters to him and his reply,—which he attributes partly to the necessity he found himself under of making fresh experiments to elucidate the subject in dispute, proceeds as follows:

"Of the three letters which you have addressed to me, the first and the third contain reasonings against the propositions I have maintained in my memoir; the second contains new experiments, which you believe to contradict mine, but which, in fact, do not do so. I could, and perhaps I ought to, content myself with replying to the second letter alone, because our dispute entirely depends upon experiments, and the most subtile reasonings will avail nothing against these: for if, from experiment, it is found that the ligature, taken away on the third or fourth day, causes the division of the artery on the following days, all arguments to prove the contrary become useless; and if, by the force of extraordinary ingenuity, it is possible to obscure the question, so as to make that appear reasonable which is contrary to what experiment proves, our conclusion must be that such reasoning is erroneous. But the regard and respect which I owe you on so many accounts, demands that I should reply to your objections one by one; and which objections, although not really just, bear the stamp of that genius which dictated them.

"You begin your critical reflections by condemning me for having asserted, *that an artery, being exposed to the action of the air, and to that which results from the operation necessary to tie it, and also to the irritation of the ligature for four days, it is no wonder if it inflames and goes into suppuration.* You condemn this proposition, because the ligature of the great vessels is performed quickly, in consequence of which the artery is exposed for a very short time to the action of the air; and the irritation is trifling, because the pressure which is made by the ligature round the artery, with the intervention of the cylinder or compress, does not exceed that which is necessary to place the parietes of the vessel in contact, and which is not made round the whole circumference of the vessel, nor upon both the vascular strata. This truth is proved, you add, by observing the adhesive inflammation to take its rise from the internal coat of the artery, at the spot where the ligature was tied; a process salutary and not morbid, and which could not happen if the ligature produced serious and inevitable mischief to the life of the vessel so tied. You add, moreover, as a thing demonstrated, that the ulceration and death of the vessel form a secondary and successive process to that of the adhesive inflammation; a process which it is in the power of art to arrest, because occasioned by the protracted presence of the ligature; and, although the point of the ligature upon which the compress rests should even form a superficial eschar, there would be no reason to believe, on that account, that, when the ligature is taken away, this eschar would not separate and fall off, as all other eschars do."

To all this M. Vacca replies, and we think very reasonably, that, however skilful in anatomy and dextrous in the use of his instruments a surgeon may be, that laying bare a large arterial trunk, tying it, and keeping a compress and ligature in a wound for some days, must necessarily occupy a certain degree of time, and produce *some* irritation;—that the placing the sides of the artery merely in contact does not, in fact, subtract one atom from the compression made upon it, nor does the interposition of the cylinder lessen that compression; the only difference being, that it is *immediate* on the posterior part of the vessel, and *mediate* on the anterior: "*but still it is compression.*" It is not necessary entirely to interrupt the circulation of blood in a vessel, to produce inflammation and suppuration; an irritation kept up for a certain length of time is quite sufficient for this purpose: and it is not to be wondered at if, although adhesive inflammation should in the first instance be excited, that suppuration and ulceration should follow. "Do we not (says M. Vacca,) see the same thing occur in the simplest wounds, which have not been irritated by dissection, nor by the presence of foreign bodies, and which display some slight adhesions, and afterwards suppurate abundantly?" (p. 7.) To illustrate still further the necessity of the occurrence of suppuration in such wounds, he adduces the familiar instance of a thorn which has remained in the flesh for three or four days, and which, although then extracted, will not prevent the parts from inflaming and suppurating, in a great majority of cases.

To Scarpa's second objection, which is couched in terms expressive of his astonishment that any one should doubt the permanent obliteration of an artery at the point where it is tied, provided the ligature has remained in its situation for four days, our author replies:

"My doubts would appear reasonable, although unsupported by those observations and experiments which convert my doubt into mathematical certainty. If you will give yourself the trouble to put together all the existing observations, dispersed in various works and Journals, on this particular point, it is most certain that these would be more than sufficient to demonstrate that, at the epoch above mentioned, the coats of the artery are found to be sufficiently re-united to resist the rush of the blood; and this proposition has never been impugned by me. But, sir, a much greater number of observations than you could ever bring together would not be able to show the *impossibility of union not taking place*, if that be true, which I conceive to be proved beyond a doubt,—namely, that inflammation in different individuals, though produced from the same cause, may develop itself sometimes earlier and sometimes later.

"But I go farther. Facts are not wanting, from which it results that the arterial parietes in the human subject, although healthy, are not united after having been tied for four days. M. Dupuytren, in the dissection of a man, aged forty-two, who died after the operation of ligature on the carotid artery, the sixth day after the operation, found the following appearances:—All the parts comprised in the operation were in that state of inflammation which confounds all the tissues, augments their density, and diminishes their power of resistance. The ligature comprised only the artery and a few fibres of the sterno-hyoideus; there was neither coagulum nor blood in the interior of the vessel; the point which was tied was puckered up, and had become so contracted that it was impossible to restore its natural calibre without tearing it.—It is true (continues M. Vacca,) that this man had suffered from violent hemorrhages before the ligature of the carotid, but still the parts had been susceptible of inflammation; and, as it was the sixth day,—that is, the period which, at the very latest, you admit of the action of the ligature,—it is clear that the blood would at that period have passed through the vessel, notwithstanding its presence.

"Hodgson details another interesting case, which applies excellently to our purpose. In an elderly man, who had suffered a very great loss of blood, the external iliac artery was tied: at the termination of the fourth day, the man died. The dissection of the body showed the ligature in its situation, without inflammation externally, and without any union between the coats of the artery, although they had been completely divided by the ligature; a method which causes inflammation to develop itself rather more quickly. Are you of opinion that, in this case, in which, from the age of the patient and the hemorrhage he had suffered, the removal of the ligature ought, according to your opinion, to have been deferred for two days longer only; that those two days would have been sufficient to have lighted up the adhesive inflammation, and to render the adhesions sufficiently firm to resist the flow of the blood? And, if you admit that it is necessary to retard the removal of the ligature to the sixth day, because old age, weakness, and other causes unknown, may delay the formation of the adhesions and coagula, how can you establish, with mathematical precision, that this union may be delayed to the sixth day, and no longer?" (p. 10.)

Our author fairly admits that he cannot look for a favourable result, if any serious disease exists in the coats of the artery; but he does not fear the longer continuance of the ligature, because it has already done all the mischief it is capable of doing in such cases; and as these cases cannot generally be distinguished *a priori*, leaving the ligature is of the greatest use in the one instance, and a matter of indifference in the other. In confirmation of this opinion, our author relates the following case:

"A man, of the name of Caparrini, had his crural artery tied, in the hospital of Santa Maria Nuova, at Florence, for the cure of a popliteal aneurism. At the beginning of the fourth day, the ligature was removed. No symptoms, to give uneasiness as to the result, came on for several days; the circulation by the collateral branches was established; there was no inflammation of the limb: but a fever came on, which was at first considered as gastric, and afterwards as a low nervous fever, and the patient died on the twenty-fifth day after the operation. The examination of the body proved satisfactorily, to all present, the ulceration of the artery on the posterior and internal surfaces, and which had, at that point, consumed the entire coats of the vessel; the iliac arteries and the aorta not appearing to be thickened. But, even granting that in this case there was a diseased condition of the arterial system, this observation shows that you have been mistaken in reckoning upon the artery not being cut through when the ligature is removed, the vessel being in a diseased state."

The above case is the more valuable as it occurred in the practice of a firm partizan of Scarpa's opinions.

In allusion to a case recorded by Sir ASTLEY COOPER, Vacca observes as follows:

"We take very different views of this (Cooper's) observation: the blood which passed through the vessel thirty-two hours after it had been tied, proves that period not to have been sufficient to form adhesions or coagula strong enough to arrest the current of blood. The blood which *did not pass* forty-eight hours after the application of the ligature, proves that the adhesions, &c. were then strong enough to put a stop to the circulation through the vessel. The hemorrhage which appeared on the twelfth day, proves that the coats, which were not ruptured on the fourth day, gave way after the ligature was removed; or that the adhesions and coagula, which governed the impetus of the blood in the first days, were at length overcome by the force of the circulation. Reflect, now, that if the artery had been in the diseased condition that you imagine, and had not felt the stimulus of the ligature, the blood would have continued to pass by the part tied after the forty-eight hours; and if the ligature produced the death of the vessel, as it was removed on the third day, it is clear that death may ensue even when the thread is removed so early; and which is precisely what I affirm, and what you deny." (p. 13.)

After obviating some objections that M. Scarpa had made to his reasoning, and which implied an apparent contradiction, our author continues:

"Permit me to examine what you have written relative to the band of lymph which surrounds the artery above and below the ligature, as well as at the point tied when the thread has fallen off. This band may certainly serve to bind and strengthen the artery both above and below the ligature, as well as at the point tied, when the cicatrix is formed; but it is not sufficient alone to oppose an hemorrhage, because, around the part which has been embraced by the ligature, the band (of lymph) is not formed until after the ulceration of the parieties of the artery, and at the period of the formation of the cicatrix. In different animals which I have made the subjects of experiments, I have not found the lymph, but suppuration, round the spot compressed by the ligature, if I examined them before the cicatrices had formed."

We agree, also, with M. Vacca in considering this removal of the ligature as not so slight an affair as Scarpa would induce us to believe; and the publication of a memoir by MAZZONI on this subject confirms us in the belief; for from this it appears that, although originally a supporter of that opinion, he was induced to abandon it, from the pain and difficulty attendant upon the operation. With regard to the rapid recovery of those patients from whose arteries the ligature has been removed on the third or fourth day, our author denies the fact; and, from his experiments, he concludes that, when this is done, suppuration always follows. "I do not deny (he says,) that excessive suppuration sometimes takes place round the ligature; that it becomes protracted, and insinuates itself into the cellular membrane of the artery, as well as that of the muscles; and that, in consequence, obstinate secondary attacks take place upon the spot where the ligature had been placed. These unpleasant events are not, however, always the consequence of the ligature, and still less of its being left beyond the fourth day. Evils of this kind occur, as you well know, in wounds where no ligatures have been used, and in which there are no foreign bodies." (p. 18.) And here he quotes Scarpa's own opinion from his work on Aneurism, and which substantially admits this fact in its full extent. He acknowledges the inutility of the ligature in those cases in which the arteries themselves are diseased; but where there is only simple atony of the coats of the vessel, prolonging the sojourn of the ligature may be beneficial: such cases cannot be distinguished *a priori*, and that method, therefore, will not be hurtful in any instance, and may be of real use in other cases; and, whatever may be the fate of our theoretical views, it will always remain true, that if you meet with an individual in whom the adhesive process has been tedious, the blood will repass by the vessel; if you meet with a case in which, either from a total or partial want of sufficient vitality, inflammation, as soon as it appears, takes on the suppurative or ulcerative stages, (the causes which excited it remaining,) then suppuration or ulceration will not be avoidable; for the experiments made upon animals, and the observations on the human subject, incontestibly prove that the action of the ligature continued for four days upon an artery, produces a condition of its coats which generally conduces towards ulceration when they are in a sound state, and certainly much more so when they are diseased. If the state of the fluids can, as both parties agree, retard or accelerate the formation of coagula and adhesions, this forms an additional reason for suffering the ligature to remain. Finally, our author observes that he follows the opinion of many other surgeons, in believing that consecutive hemorrhage is more frequent after aneurism than amputation, because the diseased condition of the vessel is more common in the first than in the second case.

We now come to the consideration of M. Vacca's second letter, and which contains the details of his experiments chiefly. He begins by lamenting that M. Scarpa did not repeat his (Vacca's) experiments, by which means any errors in their results might have been noticed, instead of forming conjectures as to the probable causes of the difference between their respective observations: in consequence of this, he is induced to give the following account of some fresh experiments which he performed.

"Although (he continues,) I had taken every precaution in my first experiment not to draw the ligature too tight, but, as I found that M. Panizza, in those which he performed, did not tighten the ligature so much as to put the parietes of the artery even into *close contact*, (as all surgeons have directed, and as you have taught, both in your work on Aneurisms, and in your memoir on Ligatures,) I was determined to follow the plan of the above-mentioned gentleman.

"My first experiment was performed on a large dog, about six years of age. I tied both the crural arteries. I drew the knot so lightly, that some of the assistants could not determine whether the circulation was entirely interrupted or not. On the fourth day, the wounds were suppurating: from the left side the ligature was removed; it was suffered to remain on the right side. Not a drop of blood was lost from either side; but, twenty-nine hours after the excision of the ligature, a hemorrhage of arterial blood appeared from the part from whence the ligature had been removed, which lasted about an hour, moderate in the beginning, and at length becoming trifling, and which ceased without any assistance. The day after, a fresh hemorrhage made its appearance from the same part, much less in degree than the former, and which spontaneously and quickly ceased. The first loss of blood was calculated at six ounces, and the second at two. Five days after the removal of the ligature, the dog was killed. In the dissection, I found that the wound was in a state of suppuration, from the part whence the ligature had been removed, though not extensively; its edges were not swollen; the inguinal glands were somewhat enlarged. On laying bare the artery, I found it surrounded by suppuration, both above and below the point where the ligature had been; and I found the lymph, as usual, surrounding the vessel. On the spot where the cylinder, or compress, had rested, the artery was of a black colour. On tearing it open longitudinally, I found that the black point was a coagulum of blood, which lay over the erosion of the anterior part of the vessel, which was entire in all its other parts upon which the ligature had acted. On the right side, where the ligature remained, I found the same appearances, including the erosion of the anterior parietes, and the clot of blood that covered it; which in this case was not moved, (mark well,) the compress which corresponded with it not having been taken away." (p. 23.)

The second experiment is still more important: we shall abridge it. Both the crural arteries of a middling-sized dog were tied in the usual manner; the knot was not drawn tight; and the edges of the wound were brought together with adhesive plaster. At the end of the third day, on attempting to cut away the ligature, a little blood appeared. Supposing that the artery was cut through, the dog was immediately killed, in order to ascertain the state of the parts. The artery was found not to be cut through: the bleeding came from a small branch. There was one small clot of blood above the knot; the parietes of the part tied were not adherent, and they presented less resistance in this place than in other parts of the vessel, and a less force was necessary to break them.

From these experiments, our author concludes that the arterial coats may be cut through without drawing the ligature very tight, and that the compress and ligature may serve to maintain the coagulum in its situation, and to avert the danger of hemorrhage.

To clear up other doubts, the following experiments were instituted:—A sheep, of large size, and aged, had the carotid and crural arteries of the same side tied. On the fourth day, the ligatures were removed from both the vessels: the edges of the wounds were scarcely swollen. Five days afterwards, the sheep was killed, and the body examined. The wound in the throat was much lessened, and covered with a dry scab, under which there was a trifling quantity of matter. Above and below the part tied, the coagulable lymph was found surrounding the artery; the part where the ligature had been was surrounded by matter. On opening the vessel longitudinally, both above and below the part tied, the usual coagula were found, the upper one of greater length than ordinary, both adhering very firmly to the sides of the vessel. These coats, at their posterior parts, and exactly where the ligature had acted, were corroded; but the close adhesions of the coagula had prevented the escape of the blood. The crural artery presented precisely the same appearances, but there was no erosion in any part of it: it was, however, evidently less firm in the point where it had been tied than natural, and was torn more easily than in other parts.

The right carotid artery of a broken-winded mare, twelve years of age, was tied as usual; the wound was closed by straps and sutures. On the fourth day the ligature was removed, and the straps re-applied. On the thirteenth day, reckoning from the day when the artery was tied, the wound was much lessened, but not healed: it still suppurated, and the mare was killed. The matter was found chiefly at the spot where the knot had been placed: neither the jugular vein nor the eighth pair of nerves were comprehended in it. Both above and below the ligature there was much lymph, which united it to the jugular vein and to the adjacent parts. The artery immediately under the compress presented a black spot, which, upon opening the vessel longitudinally, was found to be formed of a thick clot of blood, evident to the eye of the examiner; for in this point the artery was evidently corroded. This coagulum extended itself a great way, both above and below the part tied; and was single, and not double, as in all the preceding instances. Strong adhesions were formed on all points of the arterial parietes. This disposition of the coagulum, a novelty to M. Vacca, convinced him that the knot, in this case, had not been drawn tight enough to place the sides of the artery in contact. The internal coat of the vessel, in the neighbourhood of where the ligature had been, was of a dark colour, and was more easily ruptured than the parts in the vicinity.

Our author is convinced, from the above experiments, that the difference between the results of those of Scarpa and his own neither arose from the different size of the animals, from the different structure of the arteries, nor from the degree of tightness of the ligature; but from the examination of the bodies, in the experiments of M. Scarpa, having been made either too late or too soon,—that is, either before the division of the vessel has taken place, or when cicatrization has so far advanced as not to permit us to see, or to decide with certainty upon the state of the parts. To clear up this doubt, he made the following experiments:

The right carotid artery of a young and robust sheep was tied in the usual manner; the knot was very loosely tied. The crural artery of the same side was secured in the same manner. In the neck, the wound was brought together with adhesive straps; that in the thigh with sutures. The sheep went on well for three days: on the fourth, it was seized with pains in the belly, and died of mortification of the intestines. On examining the arteries, the usual appearances were found: the parietes of the artery were not corroded; the internal coat only was a little tumefied, and somewhat softened on the spot which had been tied. Immediately under the knot, the sides of the vessel were not in perfect contact; a small clot of blood placed between them served to unite the superior and inferior coagula together.

The following quotation we give in M. Vacca's own words, and with it we shall conclude our analysis of this little work, which we have extended to this length, partly on account of the importance of the subject, and partly because we believe the work is not much, if at all, known in this country.

"To convince myself (says M. Vacca,) more thoroughly of the impossibility of distinguishing, after the cicatrix is formed, whether an artery has been divided, and has afterwards united, since it is then surrounded by much lymph, which has become solid, and which binds and confounds it with the neighbouring parts, I submitted a lively dog, of two years of age and of middling size, to the following experiment:—I tied the carotid and crural arteries of the same side in the usual manner. On the fourth day, the ligatures fell off from the neck and thigh; an evident proof of the recision of the arteries. At the end of twenty-one days, the wounds were entirely healed. At this period I tied the right crural artery, and removed the ligature on the third day: the artery was entire. After eighteen days, this wound was also healed. I caused the dog to be killed two days after the perfect cicatrization of the parts, and I requested the dissector of our university to make the examination of the arteries that had been tied, in my presence. The following were the results of this examination:—The cicatrix was adherent to the parts beneath. Much hardened lymph was effused round the part which had been tied, and extended about the breadth of eight lines above and below this spot. This lymph bound the carotid artery and the eighth pair of nerves firmly together. The dissector succeeded, by dividing this lymph longitudinally, in separating it into two cords; one of which corresponded to the artery, the other to the nerve. These two portions appeared perfectly entire, and not interrupted in any part of their continuity. The artery was obliterated to the distance of a few lines from the part which had been tied, at its lower extremity; at the upper part, the obliteration extended somewhat farther: the whole distance together obliterated amounted to about eighteen lines. The coagulum did not exist at the lower extremity; it was found at the upper, and was even still coloured. With a very fine probe, the dissector removed the substance which filled the cavity of the artery, and which had become a cord: he laid open the vessel longitudinally, and none of us could determine the spot where this cord had been divided by the ligature. There could be no doubt of this division having taken place, as the ligature had fallen off spontaneously. The same appearances presented themselves on examining the crural artery of the same side. The right crural artery, from which the ligature was removed on the third day, appeared, as usual, surrounded by a good deal of coagulable lymph, confused with the neighbouring parts; but, on separating this lymph, (which, being more recently formed, had less tenacity and consistence than in the other instances,) it was clearly seen that a portion of this lymph had insinuated itself into an aperture in the coats of the artery, and which was made apparent as soon as the lymph was removed with the forceps. This lymph had formed strong adhesions to the sides of the vessel, but they were not yet so strong as to be inseparable.

"From this experiment it is apparent, that, although arteries are cut through by the ligature, they re-unite, after a certain time, in such a way, and are so surrounded by lymph, that it is not possible to distinguish the spot where the division has been; and that arteries, from which the ligatures have been removed on the third day, present the same appearances: for it was entirely by accident, and from not having given the lymph time to consolidate, that we were able to discover the erosion of one portion of the parietes of the vessel. If the examination had been delayed a few days, it would have been impossible to have found any traces of erosion."

---

### STATISTICAL MEDICINE.

